# The Effects of Endoscopic Third Ventriculostomy Versus Ventriculoperitoneal Shunt on Neuropsychological and Motor Performance in Patients with Idiopathic Normal Pressure Hydrocephalus—ENVENTOR-iNPH: Study Protocol

**DOI:** 10.3390/brainsci15050508

**Published:** 2025-05-16

**Authors:** Gianluca Scalia, Nicola Alberio, Pietro Trombatore, Mariangela Panebianco, Grazia Razza, Gianluca Galvano, Giovanni Federico Nicoletti, Francesca Graziano

**Affiliations:** 1 Unit of Neurosurgery, Department of Head and Neck Surgery, Garibaldi Hospital, 95124 Catania, Italy; nicolettigiovanni23@gmail.com (G.F.N.); fragraziano9@gmail.com (F.G.); 2 School of Medicine and Surgery, Kore University of Enna, 94100 Enna, Italy; 3 Department of Neurosurgery, Cannizzaro Hospital, 95124 Catania, Italy; nickwam1971@gmail.com; 4 Department of Diagnostic Imaging, Interventional Radiology and Neuroradiology, Garibaldi Hospital, 95124 Catania, Italy; pietro.trombatore@gmail.com (P.T.); gianlucagalvano@gmail.com (G.G.); 5 Unit of Neurology, Garibaldi Hospital, 95124 Catania, Italy; mariag.panebianco@gmail.com; 6 Department of Mental Health, ASP Catania, 95124 Catania, Italy; grazia.razza@aspct.it

**Keywords:** idiopathic normal pressure hydrocephalus, ventriculoperitoneal shunt, endoscopic third ventriculostomy, randomized controlled trial, nTMS

## Abstract

**Background:** Idiopathic normal pressure hydrocephalus (iNPH) is a progressive neurological disorder characterized by cognitive decline, gait disturbances, and urinary incontinence. Surgical interventions such as ventriculoperitoneal shunt (VPS) and endoscopic third ventriculostomy (ETV) are the primary treatment options. While VPS is the standard of care, ETV offers a minimally invasive alternative with potentially fewer complications. However, comparative evidence regarding their impact on cognitive, motor, and structural outcomes remains limited. This study, titled ENVENTOR-iNPH (endoscopic ventriculostomy versus shunt on neuropsychological and motor performance in patients with iNPH), aims to address this gap through a rigorously designed comparative protocol. **Methods:** This protocol is designed as a multicenter, randomized, controlled trial (ENVENTOR-iNPH) to compare the effects of ETV and VPS in patients diagnosed with iNPH. The study will enroll 100 patients aged 60 years or older, randomly assigned to undergo ETV (n = 50) or VPS (n = 50). Preoperative and postoperative evaluations will include comprehensive cognitive and motor assessments, standardized quality-of-life instruments, and advanced neuroimaging techniques such as MRI with flowmetry and diffusion tensor imaging (DTI). Functional outcomes will also be evaluated using navigated transcranial magnetic stimulation (nTMS) and wearable motion analysis systems. The objective of this study is to compare the efficacy and safety of ETV versus VPS in restoring cognitive and motor performance in patients with iNPH. **Results:** Primary outcomes include cognitive and motor function improvements. Secondary endpoints are surgical complications, hospital stay duration, and changes in quality of life. Neuroimaging will assess changes in white matter integrity and cerebrospinal fluid dynamics, while nTMS will provide insights into neuroplasticity and motor pathway recovery. ETV is hypothesized to demonstrate clinical outcomes comparable or superior to VPS, particularly in terms of complication reduction and hospital recovery metrics. **Conclusions:** The ENVENTOR-iNPH protocol establishes the framework for a comprehensive, multicenter study comparing ETV and VPS in iNPH patients. The findings from this initial study will inform the design of larger-scale multicenter trials, guide clinical decision making, and potentially position ETV as a preferred treatment option for eligible patients.

## 1. Introduction

Idiopathic normal pressure hydrocephalus (iNPH) is a chronic neurodegenerative disorder that primarily affects older adults. It is characterized by Hakim’s triad of symptoms: cognitive decline, gait disturbances, and urinary incontinence [1,2]. Unlike other forms of hydrocephalus, iNPH occurs without an increase in intracranial pressure (ICP), yet it leads to progressive ventricular dilation. The exact pathophysiology remains unclear, but it is believed to involve impaired cerebrospinal fluid (CSF) absorption, resulting in ventricular expansion and compression of periventricular white matter tracts [1,2,3]. This compression disrupts the neural pathways responsible for cognitive, motor, and urinary control, thereby explaining the hallmark symptoms. Given the clinical overlap with Alzheimer’s and Parkinson’s diseases, iNPH is frequently misdiagnosed, delaying timely intervention.

Surgical treatment is the most effective approach for managing iNPH, with ventriculoperitoneal shunt (VPS) and endoscopic third ventriculostomy (ETV) being the two most widely used techniques [4,5,6]. VPS involves diverting excess CSF from the ventricles to the peritoneal cavity using an implanted device, thereby relieving ventricular pressure. While effective, VPS carries risks of infection, device malfunction, and over-drainage, often requiring shunt revisions [7]. By contrast, ETV creates a direct flow path for CSF from the third ventricle into the subarachnoid space, bypassing the need for an implantable device. ETV reduces the risk of infection and device-related complications but is dependent on favorable patient anatomy and surgical precision [8].

Both procedures are effective in alleviating the symptoms of iNPH, with gait improvement often seen as the earliest and most prominent benefit [9]. However, the impact on cognitive recovery is less consistent, with prior studies suggesting that early surgical intervention leads to better cognitive outcomes. While previous research has demonstrated the efficacy of ETV and VPS independently, direct comparative studies are limited, and the relative long-term effectiveness of these techniques remains underexplored.

To address this gap, we have developed the ENVENTOR-iNPH (endoscopic ventriculostomy versus shunt on neuropsychological and motor performance in patients with iNPH) study protocol. This acronym reflects the central focus of the study, as also emphasized typographically in the manuscript title, where ENVENTOR-iNPH is distinguished to signal its importance as the study protocol name.

This study aims to provide robust evidence on the comparative cognitive, motor, and structural outcomes of ETV versus VPS using advanced neuroimaging, diffusion tensor imaging (DTI), and navigated transcranial magnetic stimulation (nTMS). By leveraging these tools, this study will offer new insights into the mechanisms of recovery and inform clinical guidelines for iNPH treatment. Although this initial protocol excludes patients with significant neurological comorbidities to maintain internal validity, we acknowledge this may limit generalizability. Future phases of the ENVENTOR-iNPH project will aim to include stratified cohorts with mild or early-stage comorbid conditions and potentially utilize biomarker-supported diagnoses to enhance translational relevance.

## 2. Materials and Methods

This study is structured as a randomized, controlled, multicenter clinical trial designed to compare the effects of ETV and VPS on the cognitive, motor, and structural outcomes of patients with iNPH. To ensure appropriate patient allocation between the ETV and VPS arms, standardized clinical and radiological criteria have been established. These include a positive response to the tap test (TT), the presence of gait disturbance, mild-to-moderate cognitive impairment, and radiological findings such as an Evans index > 0.3, callosal angle < 90°, and DESH. MRI with flowmetry will be used to assess anatomical suitability for ETV, particularly evaluating the configuration of the third ventricular floor and patency of basal cisterns. These uniform criteria will be used across all participating centers to ensure methodological consistency in this multicenter trial, which is aligned with the recently proposed six-step diagnostic and therapeutic algorithm endorsed by the Italian Society of Neurosurgery (SINCH) for the management of iNPH [10]. The trial will span a period of 36 months and involve multiple healthcare centers, ensuring a diverse and representative patient population to enhance the generalizability of the findings. The study protocol is named ENVENTOR-iNPH (endoscopic ventriculostomy versus shunt on neuropsychological and motor performance in patients with iNPH). A total of 100 patients diagnosed with iNPH will be enrolled and randomly assigned in a 1:1 ratio to undergo either ETV or VPS. Randomization will be performed using a computer-generated algorithm, ensuring allocation concealment and reducing the risk of selection bias. Eligibility criteria require participants to be 60 years or older with a confirmed diagnosis of iNPH. This diagnosis must be established through a combination of clinical neurological assessment, invasive diagnostic testing, and neuroimaging criteria. Invasive tests include the TT, which evaluates clinical improvement following CSF removal, and the LiquoGuard test, a continuous CSF monitoring and drainage procedure that provides detailed intracranial pressure and flow measurements. Neuroimaging will play a critical role in diagnosis and treatment planning, utilizing MRI and DTI to assess ventricular enlargement, periventricular white matter changes, and CSF flow dynamics. MRI with flowmetry will measure the velocity and dynamics of CSF circulation, aiding in confirming the diagnosis and determining the anatomical suitability for ETV. Patients will also undergo computed tomography (CT) imaging as part of the preoperative assessment to evaluate ventricular anatomy, assess for calcifications, and exclude contraindications such as significant basal cistern obstructions or intracranial lesions. Neuroimaging will also assess the presence of DESH (disproportionately enlarged subarachnoid space hydrocephalus), defined by widened Sylvian fissures, narrowed high-convexity sulci, and ventriculomegaly. Together, these diagnostic tools will provide a comprehensive evaluation of the patient’s condition, ensuring accurate diagnosis and optimal patient selection for the study. Exclusion criteria include the presence of comorbid neurological conditions, such as Alzheimer’s disease or Parkinson’s disease, which may confound outcome measurements, as well as anatomical variations (e.g., severely narrowed third ventricle floor or basal cistern scarring) that would render ETV technically unfeasible. Additionally, patients unable to provide written informed consent or who exhibit contraindications to invasive diagnostic tests will be excluded from participation. This rigorous approach to patient selection, encompassing both advanced neuroradiological imaging and invasive diagnostic testing, ensures a homogenous study population and enhances the reliability and validity of the trial’s outcomes.

### 2.1. Preoperative Evaluation

**Figure 1:**
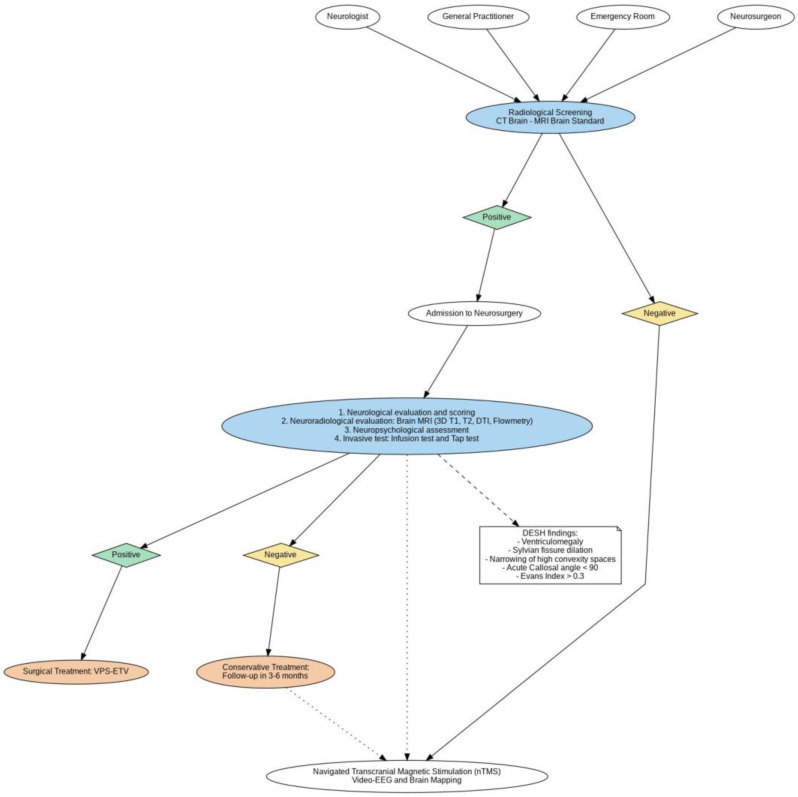
Flowchart illustrating the diagnostic and treatment pathway for idiopathic normal pressure hydrocephalus (iNPH), starting with referrals and radiological screening (CT/MRI). Based on the results, patients either undergo advanced evaluations in neurosurgery or exit the process if the findings are negative. Further steps involve neurological, neuroradiological, and neuropsychological assessments, along with invasive testing. Positive findings lead to surgical interventions like VPS or ETV, while negative results suggest conservative treatment with follow-up. The chart also highlights the criteria for iNPH (e.g., DESH findings) and supplementary options like transcranial magnetic stimulation and brain mapping.

Preoperative evaluation is critical for establishing a detailed baseline of each patient’s neurological, cognitive, motor, and personality functions. It combines clinical assessments, advanced neuroimaging, and specialized tests to confirm the diagnosis and plan the surgical approach (Figure 1). All neuropsychological assessments, including WAIS-IV, RBMT-3, Raven’s matrices, and MMPI-2, will be administered preoperatively and repeated at each follow-up to allow longitudinal comparison. Motor assessments, including the Timed Up and Go (TUG) Test, Baiobit wearable sensor gait analysis, and the iNPH grading scale, will also be conducted both preoperatively and at each postoperative timepoint.

### 2.2. Neurological Examination and Scoring Systems

A detailed neurological examination will assess motor, cognitive, and sensory functions, with a particular focus on hallmark symptoms of iNPH, such as gait disturbances and reflex abnormalities. Objective tools include the following:

- Motor test: Timed Up and Go (TUG) Test, 6 min walk through the Baiobit system, a wearable inertial measurement unit (IMU) to measure precise balance, mobility, and fall risk [4].
- iNPH grading scale (iNPHGS): a validated tool to quantify the severity of gait, cognitive, and urinary symptoms, ranging from 0 (no symptoms) to 4 (severe impairment) [11].
- Kiefer score: integrates functional symptom severity with radiological findings, including ventricular enlargement and disproportionate subarachnoid spaces measured using the Evans index [12].

### 2.3. Neuropsychological Assessment

A series of standardized neuropsychological tests will evaluate the cognitive domains commonly affected in iNPH, providing critical data on intellectual, memory, and personality functioning:

- Raven’s progressive matrices to measure non-verbal intelligence and assess fluid reasoning and problem-solving abilities independent of cultural or educational background 4.
- Rivermead Behavioural Memory Test—Third Edition (RBMT-3), a memory test with ecological validity designed to predict memory deficits in real-life scenarios and monitor changes over time [12].
- WAIS-IV (Wechsler Adult Intelligence Scale—Fourth Edition) to provide a comprehensive evaluation of cognitive abilities, including general intellectual functioning, working memory, fluid intelligence, and processing speed [4,12].
- Minnesota Multiphasic Personality Inventory—2 (MMPI-2) to assess personality traits and emotional disorders, offering insights into psychological factors that may influence postoperative recovery [11].

In addition to these instruments, the Mini-Mental State Examination (MMSE) will be used to provide a rapid, global assessment of cognitive function. To evaluate balance and fall risk, the Berg Balance Scale (BBS) will be administered, and the Modified Rankin Scale (mRS) will be employed to assess overall functional independence and the degree of disability.

### 2.4. Neuroimaging

Advanced imaging protocols are critical for confirming the diagnosis and guiding surgical planning. The imaging protocol included the following:

- MRI with flowmetry, which evaluates CSF flow dynamics and identifies ventricular enlargement and disproportionate subarachnoid spaces. This imaging technique is particularly useful for determining anatomical suitability for ETV [4,11].
- Diffusion tensor imaging (DTI) to assess the integrity of periventricular white matter tracts and identify the structural changes associated with ventricular compression and potential recovery [12].
- CT scans, complementing MRI by evaluating calcifications, bone structures, and basal cistern anatomy, which are important for surgical feasibility [11].

### 2.5. Neuroinvasive Diagnostic Tests

Invasive tests will be used to confirm the diagnosis of iNPH:

- Tap test (TT), which involves removing a large volume of CSF to observe symptom improvement in gait, cognition, or urinary function, offering strong predictive value for surgical success 4.
- LiquoGuard Test, which provides continuous CSF drainage and intracranial pressure monitoring, delivering detailed data on CSF dynamics to validate the diagnosis and refine treatment plans [12].

### 2.6. Neurofunctional Evaluation

nTMS will be employed to assess cortical excitability and motor pathway integrity. This non-invasive technique uses magnetic fields to stimulate motor cortical regions, allowing for the mapping of motor pathways and the recording of motor evoked potentials (MEPs). nTMS will establish a baseline for corticospinal tract functionality, which will be critical for tracking postoperative motor recovery and neuroplasticity [12].

### 2.7. Surgical Procedures

Patients will be randomly assigned to undergo either ETV or VPS. Both procedures will be conducted by experienced neurosurgeons at the participating centers using standardized techniques to ensure consistency and safety.

### 2.8. VPS Surgery

The VPS procedure involves the placement of a catheter in the lateral ventricle to drain excess CSF into the peritoneal cavity, where it is reabsorbed. A programmable valve, chosen based on the surgeon’s clinical judgment, will regulate the flow of CSF. This approach allows for adjustments in drainage settings postoperatively to optimize patient outcomes and reduce complications such as over-drainage [12].

The surgical technique will follow the standardized steps, including positioning patients in the supine position, preoperative prophylactic antibiotics, placement of a ventricular catheter under neuronavigation, connection to a programmable valve, and distal catheter tunneling into the peritoneal cavity. Postoperative valve settings will be tailored individually, and infection control protocols will be uniformly applied across centers.

### 2.9. ETV Surgery

ETV is a minimally invasive procedure in which a small perforation is made in the floor of the third ventricle, creating a new pathway for CSF flow into the basal cisterns. ETV will be performed with the patient in the supine position using a rigid endoscope. After identifying anatomical landmarks, a perforation will be made in the third ventricular floor anterior to the mammillary bodies. Fenestration patency will be confirmed visually and with irrigation. Neuronavigation and intraoperative monitoring will assist in avoiding vascular injury and ensuring procedural safety [11].

### 2.10. Postoperative Evaluation

The postoperative follow-up protocol is designed to evaluate recovery and assess the efficacy of the surgical interventions. Follow-ups will occur at 1 week, 3 months, 6 months, and 12 months after surgery. During these evaluations, cognitive and motor assessments will be repeated, and imaging studies will track the structural changes in white matter and ventricular size. At the follow-up times, patients will undergo repeated neuropsychological and motor performance evaluations using the same tools administered preoperatively. This will enable a direct comparison of cognitive and motor recovery over time [4,12]. MRI with flowmetry and DTI will be performed during each follow-up to monitor changes in ventricular size, CSF flow, and white matter integrity. These imaging modalities provide critical insights into the structural effects of ETV and VPS on the brain [11,12]. At the same follow-up, nTMS will assess postoperative changes in corticospinal excitability and motor pathway recovery. Changes in MEPs will serve as objective indicators of neuroplasticity and functional improvement. Additionally, repetitive TMS (rTMS) may be explored as a therapeutic tool to further enhance motor recovery [12]. This integrated approach ensures a thorough evaluation of the effects of ETV and VPS on iNPH patients, providing a robust dataset to inform clinical decision making and improve treatment outcomes.

Quality of life will also be systematically assessed using the SF-36 Health Survey, a validated instrument capturing both physical and mental health domains from the patient’s perspective. This tool will be administered preoperatively and at each follow-up interval to longitudinally evaluate patient-reported outcomes.

### 2.11. Statistical Analysis

All statistical analyses will be conducted using IBM SPSS Statistics, version 30.0.0, and R, version 4.5.0. The threshold for statistical significance will be set at α = 0.05. Two-tailed tests will be used unless a specific directional hypothesis is stated (e.g., the anticipated superiority of ETV over VPS), in which case one-tailed tests will be applied.

Continuous variables will first be tested for normality using the Kolmogorov–Smirnov test, and for homogeneity of variance using Levene’s test. If assumptions of normality and homoscedasticity are met, parametric tests such as the independent-samples *t*-test will be used. For repeated measures data, linear mixed-effects models will be applied instead of a traditional ANOVA to account for intra-subject variability and missing data points. If assumptions are violated, appropriate non-parametric alternatives (e.g., Mann–Whitney U test, Wilcoxon signed-rank test) will be applied.

To control Type I errors in multiple comparisons, a Bonferroni correction will be applied where appropriate. In addition to *p*-values, effect sizes (e.g., Cohen’s d for *t*-tests, η^2^ or partial η^2^ for ANOVA) will be reported to quantify the magnitude of observed effects.

For the analysis of longitudinal data, mixed-effects models will be used to account for intra-subject variability across follow-up points. These models will include fixed effects for time and the treatment group, as well as random intercepts to capture individual-level variability.

An a priori power analysis was performed assuming an effect size of Cohen’s d = 0.5, power (1 − β) of 0.80, and α = 0.05 (two-tailed). The estimated required sample size was 51 participants per group. This effect size was selected as a conservative estimate based on previously published studies evaluating pre- and postoperative cognitive and motor changes in patients with iNPH. Nonetheless, we recognize that this estimate may be optimistic. If the true effect is smaller, a larger sample size would be needed to retain adequate statistical power. This limitation is acknowledged, and future extensions of the study may involve adaptive or sequential design strategies to account for observed variability.

## 3. Expected Results

The results of this study will offer valuable insights into the comparative effectiveness of ETV and VPS in treating patients with iNPH. Cognitive, motor, and structural changes will be analyzed at the preoperative baseline and at 1 week, 3 months, 6 months, and 12 months post-surgery. Data will be collected from 100 patients who will be randomly assigned to either the ETV or VPS group. The analysis will focus on how each surgical approach influences cognitive recovery, gait improvements, and white matter integrity.

The primary outcome will be the change in cognitive and motor performance over time. Cognitive functions will be evaluated using neuropsychological tests, focusing on memory, attention, executive function, and language. All cognitive assessments will be conducted both preoperatively and at each postoperative follow-up interval, as stated in the protocol. Motor outcomes will be assessed through gait analysis and coordination tests. All motor assessments will likewise be performed preoperatively and postoperatively using standardized tools including the Timed Up and Go (TUG) Test, the Baiobit wearable system, and the iNPH grading scale. The preliminary expectations are that patients in both the ETV and VPS groups will experience significant improvements in gait, as gait disturbance is typically the first symptom to improve following iNPH surgery. However, the degree of cognitive improvement may differ between the two groups, as prior evidence suggests that early intervention and reduced white matter compression may promote better cognitive recovery. By comparing cognitive trajectories in the ETV and VPS groups, this study aims to determine whether ETV has comparable or superior cognitive benefits relative to VPS. Structural changes in the brain will be analyzed using diffusion tensor imaging (DTI) and magnetic resonance imaging (MRI) with flowmetry. DTI will track changes in white matter integrity over the 12-month follow-up period. Since periventricular white matter is particularly vulnerable in iNPH, changes in the integrity of white matter tracts, such as the corticospinal tract, will be examined. The integrity of these tracts is essential for motor coordination and cognitive functions. Tractography will be used to visualize white matter pathways before and after surgery, providing insight into how ETV and VPS differentially affect white matter recovery. Neurophysiological changes will be measured using navigated transcranial magnetic stimulation (nTMS). This technique will allow researchers to assess motor cortical excitability and map changes in corticospinal tract connectivity. Motor evoked potentials (MEPs) will be recorded before surgery and at all follow-up intervals. An increase in MEP amplitude after surgery would suggest improved cortical excitability and the potential recovery of motor function. Differences in MEP amplitudes between the ETV and VPS groups may offer additional evidence regarding which procedure promotes superior motor recovery. Secondary outcomes will include the rate of complications, intensive care unit (ICU) stay, and patient-reported quality of life. Patients in the VPS group are expected to have a higher incidence of device-related complications, such as infection, shunt malfunction, and over-drainage, compared to the ETV group, which does not require the implantation of a foreign device. ICU stay and hospital length of stay will be compared between the two groups, with the hypothesis that ETV patients will have shorter stays due to the less invasive nature of the procedure. Quality-of-life measures will be obtained from patient self-reports, providing insight into the broader impact of surgery on daily living and well-being.

### Primary and Secondary Endpoints

This study’s primary outcome is the quantification of improvement in cognitive and motor performance from baseline to 12 months after VPS surgery or ETV. Secondary outcomes include the rate of postoperative complications (e.g., infection, device malfunction, or over-drainage), length of hospitalization, ICU stay, and patient-reported quality of life. To ensure that the study has sufficient power to detect meaningful differences between the two surgical groups, a power analysis was conducted. This analysis determined that a total sample size of 100 patients (50 in each group) would be required to achieve a power of 80% to detect an effect size (d) of 0.5 at a significance level of 0.05. The randomized controlled trial design, combined with standardized surgical protocols and the use of advanced neuroimaging and neurostimulation techniques, ensures that the results will be both statistically robust and clinically relevant.

## 4. Discussion

iNPH is a progressive and treatable neurodegenerative condition that significantly impacts the cognitive, motor, and urinary functions of affected individuals, most of whom are elderly [1,2,3,13,14]. The condition, characterized by ventricular enlargement without increased intracranial pressure (ICP), is associated with Hakim’s triad of symptoms: cognitive decline, gait disturbances, and urinary incontinence [15,16]. These symptoms often overlap with those seen in Alzheimer’s disease and Parkinson’s disease, leading to frequent misdiagnoses and delays in effective treatment [16,17]. As awareness of iNPH has grown, and there has been a shift toward early diagnosis and surgical intervention, which have been shown to significantly improve patient outcomes.

Two primary surgical options exist for the treatment of iNPH: VPS and ETV. Each procedure has distinct mechanisms of action, risk profiles, and potential impacts on cognitive and motor recovery. VPS has been the “gold standard” treatment for decades, with success rates reported as high as 85% [4,5,6,7]. It involves the insertion of a catheter to drain excess cerebrospinal fluid (CSF) from the ventricles to the peritoneal cavity, thereby relieving pressure on the brain parenchyma. Despite its efficacy, VPS is associated with significant drawbacks, including the risk of infection, device malfunction, and over-drainage, which may require multiple surgical revisions over a patient’s lifetime [17,18]. Moreover, the presence of an implanted device poses a lifelong risk of infection and increases the burden on healthcare systems due to the need for follow-up and device replacement.

ETV has emerged as a promising alternative to VPS. This minimally invasive procedure involves the creation of a fenestration in the floor of the third ventricle, allowing CSF to bypass any obstruction and flow into the subarachnoid space [8,9]. Unlike VPS, ETV does not require the implantation of a foreign device, which significantly reduces the risk of infection and eliminates the need for lifelong device maintenance. Several studies have reported that ETV has a lower complication rate than VPS, with fewer incidences of infection, over-drainage, and mechanical failure [19]. However, ETV has its own limitations. Its success depends on the anatomical configuration of the patient’s third ventricle and basal cisterns, as well as the technical precision of the neurosurgical procedure [20]. In cases where the third ventricle anatomy is not suitable for fenestration, ETV may be ineffective, leading to treatment failure and the need for secondary surgical interventions. Moreover, ETV may not be appropriate for all patients with iNPH, especially those with anatomical variants or those with impaired compliance of the basal cisterns [21,22]. Despite these limitations, ETV has been associated with a lower rate of long-term complications and fewer hospital readmissions compared to VPS.

This trial, formally titled ENVENTOR-iNPH (endoscopic ventriculostomy versus shunt on neuropsychological and motor performance in patients with iNPH), has been specifically designed to overcome the methodological limitations of prior studies. The acronym ENVENTOR-iNPH also appears in the manuscript title to reinforce its role as the definitive name of this study protocol.

We hypothesize that ETV will result in cognitive and motor outcomes that are comparable or superior to those achieved with VPS, while minimizing complications and hospitalization duration.

The methodological approach of this study is designed to overcome many of the limitations observed in previous research. By using a randomized controlled design with a sufficiently powered sample size of 100 patients (50 in the ETV group and 50 in the VPS group), this study will provide a statistically robust comparison of the two surgical techniques. The a priori power analysis assumed a medium effect size (Cohen’s d = 0.5), α = 0.05, and power of 80%. While this provides a practical sample estimate, we acknowledge that the true effect size may be smaller than anticipated, potentially requiring a larger cohort to maintain adequate statistical power. This limitation is recognized in the design and discussed transparently.

To ensure procedural consistency across participating institutions, all surgical teams will follow a shared and standardized operative protocol, developed collaboratively by the study investigators. Pre-trial workshops and training sessions will be organized to align technical approaches and perioperative care pathways. In addition, a central monitoring committee will oversee compliance with the protocol and conduct periodic audits to mitigate variability and ensure methodological integrity throughout the multicenter implementation.

In terms of radiological criteria, the diagnosis of iNPH and anatomical suitability for surgical procedures will also be based on the validated indices. In particular, the Evans index—defined as the ratio between the maximum width of the frontal horns and the inner diameter of the skull—will be used to confirm ventriculomegaly when greater than 0.3. Likewise, the callosal angle, measured in the coronal plane through the posterior commissure, provides additional diagnostic confidence, with angles below 90° typically associated with iNPH.

This study employs a robust and innovative methodological framework to assess the outcomes of ETV and VPS in patients with iNPH. By integrating neuroinvasive diagnostic techniques, neuromotor assessments, neurofunctional evaluations, and advanced neuroimaging, the research addresses critical gaps in the understanding of how these surgical interventions impact cognitive, motor, and structural recovery. The use of invasive diagnostic tools, such as the LiquoGuard test and the TAP test, provides crucial insights into CSF dynamics and symptom responsiveness. The LiquoGuard test, which continuously monitors intracranial pressure and CSF flow, offers a detailed profile of pressure-flow relationships, enhancing diagnostic accuracy and predicting surgical outcomes [23,24]. Similarly, the TAP test, which involves the removal of a large CSF volume, allows for direct observation of symptomatic improvements, particularly in gait and cognition, offering strong predictive value for surgical success [25,26]. Together, these tools ensure precise patient selection, minimizing the risk of non-responsiveness to surgery.

Gait disturbances, a hallmark symptom of iNPH, are central to the evaluation of surgical effectiveness. Established tools, such as the Timed Up and Go (TUG) Test, assess balance and mobility, while the 6-Minute Walk Test measures functional endurance, both of which provide critical baseline and postoperative data [27,28]. Additionally, the innovative Baiobit system, a wearable inertial measurement unit (IMU), represents a significant advancement in gait analysis. By capturing detailed kinematic parameters, such as step length, stride variability, and symmetry, Baiobit offers unparalleled precision in assessing motor function recovery. Its application in iNPH patients, a novel approach in this field, is expected to yield valuable insights into the subtleties of gait improvement following ETV and VPS [29,30,31,32].

A key innovation of this study is the integration of navigated transcranial magnetic stimulation (nTMS) to evaluate neurofunctional recovery. By mapping motor cortical areas and measuring corticospinal excitability through motor evoked potentials (MEPs), nTMS provides objective data on the recovery of motor pathways [33]. Postoperative changes in MEP amplitudes serve as markers of neuroplasticity and functional reorganization of the motor system. This approach not only enhances our understanding of surgical outcomes but also introduces a potential therapeutic avenue through repetitive TMS (rTMS), which has been proposed as a neurorehabilitation tool to improve cognitive and motor recovery following neurological injury or surgery. The inclusion of nTMS positions this study at the forefront of functional neuroscience, bridging diagnostic and therapeutic innovations.

Cognitive deficits are a significant burden in iNPH, making their assessment a critical component of this study. The inclusion of standardized tools, such as Raven’s progressive matrices, the Rivermead Behavioral Memory Test (RBMT-3), WAIS-IV, and MMPI-2, ensures a comprehensive evaluation of cognitive domains, including reasoning, memory, and personality traits [4,33,34,35]. These assessments not only provide a detailed baseline but also allow for the longitudinal tracking of cognitive recovery, offering insights into how ETV and VPS differentially impact neurocognitive functions. Advanced imaging protocols are central to both preoperative planning and postoperative evaluation. Magnetic resonance imaging (MRI) with flowmetry quantifies CSF dynamics, aiding in the confirmation of iNPH diagnosis and evaluating shunt responsiveness [36,37]. Diffusion tensor imaging (DTI) provides a detailed view of microstructural changes in white matter, focusing on tracts such as the corticospinal tract, which are crucial for motor and cognitive recovery [38,39]. By examining the integrity of periventricular pathways, DTI helps elucidate why some patients experience more effective recovery than others. Additionally, disproportionately enlarged subarachnoid space hydrocephalus (DESH) features, including widened Sylvian fissures, narrow cortical sulci, and enlarged ventricles, that are systematically evaluated to confirm the diagnosis and predict treatment responsiveness [36]. Quantitative metrics, such as the Evans index and callosal angle, further enhance diagnostic precision, with values that exceed established thresholds strongly suggesting iNPH [37,38]. Tractography, combined with neuronavigation, not only aids in precise surgical targeting but also reduces the risk of damage to critical brain regions during ETV or VPS procedures [40].

### 4.1. VPS Versus ETV: Pros and Cons

The surgical treatment of iNPH is centered around two main approaches: endoscopic third ventriculostomy (ETV) and ventriculoperitoneal shunt (VPS) [9]. Each of these procedures offers distinct benefits and limitations, making the choice of treatment highly dependent on patient-specific factors, anatomical characteristics, and clinical goals. One of the key advantages of ETV is the elimination of the need for an implantable device [41]. Unlike VPS, which relies on a catheter and valve system to drain cerebrospinal fluid (CSF) from the brain to the peritoneal cavity, ETV creates a natural internal pathway for CSF flow. This approach avoids the risk of mechanical failure, disconnection, and infection associated with implanted devices. By removing the dependency on an external device, ETV reduces the overall burden on healthcare systems, as it does not require lifelong device monitoring or replacement surgeries. Another important advantage of ETV is the reduced risk of postoperative infection. Since no foreign object is implanted, the likelihood of infection is significantly lower compared to VPS, where the presence of an implanted shunt system increases susceptibility to bacterial colonization. ETV also provides a long-term solution for many patients, as it re-establishes the body’s natural CSF flow dynamics, thereby reducing the need for future surgical revisions [42,43]. Unlike VPS, where shunt failure often necessitates additional surgeries, ETV patients are less likely to require repeat procedures. Additionally, the absence of complications related to over-drainage, which is a known risk of VPS, further highlights the stability and sustainability of ETV. Over-drainage, often seen in VPS patients, can result in subdural hematomas, but this risk is inherently avoided in ETV due to its reliance on natural flow mechanisms. Moreover, ETV has been associated with shorter hospital stays, fewer intensive care unit (ICU) admissions, and faster recovery times. Given the minimally invasive nature of the procedure, patients often experience less postoperative discomfort and a quicker return to daily activities compared to those who undergo VPS. Despite these advantages, ETV also has certain limitations that must be considered when selecting a treatment for iNPH. A critical disadvantage of ETV is its dependence on patient-specific anatomical factors [9]. The procedure requires sufficient anatomical space within the third ventricle and basal cisterns to safely create a new pathway for CSF flow. In patients with anatomical anomalies, such as a narrow or distorted third ventricle floor, ETV may be technically infeasible. Furthermore, ETV is a more technically demanding procedure that requires specialized neurosurgical skills and advanced neuronavigation technology. The surgeon must ensure that the perforation of the third ventricle floor does not damage critical adjacent structures, such as the basilar artery, as such damage could lead to severe complications, including stroke. Another significant challenge with ETV is the risk of stoma closure. Over time, the opening created in the third ventricle may close due to scarring or the accumulation of cellular debris, thereby blocking the CSF flow and necessitating additional surgical intervention. Unlike VPS, which allows for shunt adjustments, ETV does not offer a means for mechanical control or adjustment after the surgery is complete. Additionally, while ETV is generally effective for resolving gait disturbances, the evidence regarding its impact on cognitive recovery is mixed. Cognitive improvements after ETV are often slower and less predictable, as cognitive recovery depends on the restoration of periventricular white matter pathways. Delayed cognitive recovery may be attributed to the slow process of white matter reorganization following the release of ventricular compression. From a patient-centered perspective, ETV would provide a better quality of life, as it would eliminate the need for a permanent shunt and reduce the risk of repeated surgical interventions. This outcome would also have economic benefits, as VPS requires frequent monitoring, shunt revisions, and hospitalization for complications, whereas ETV offers a more definitive, one-time solution.

On the other hand, VPS offers a different set of benefits that make it a widely used approach for the treatment of iNPH. One of its most notable advantages is its applicability to a broader range of patients [9,41,42,43,44]. Unlike ETV, which requires a specific anatomical configuration to be effective, VPS can be used in almost all patients with iNPH, regardless of their ventricular anatomy. This versatility allows neurosurgeons to treat patients who would otherwise be ineligible for ETV due to unfavorable anatomical features, such as narrow ventricles or the presence of subarachnoid scarring. VPS also provides a more predictable and immediate resolution of symptoms. Many patients experience an almost immediate improvement in gait, balance, and motor coordination following the procedure [9,41,42,43,44,45]. This immediate symptom relief can have a profound impact on patient quality of life, especially in those with severe gait impairments. The procedure is well-documented, and its effectiveness has been established through decades of clinical practice and research. Neurosurgeons are highly familiar with VPS procedures, and well-established surgical protocols ensure a high degree of predictability in outcomes. This predictability extends to the management of complications as well. Since VPS systems are widely used, shunt components, valves, and catheters are readily available, and the replacement of faulty or malfunctioning components is straightforward. The design of modern shunt systems, which often includes programmable valves, allows neurosurgeons to adjust the flow rate of CSF drainage as needed, offering greater control over the treatment process. Nevertheless, VPS is not without its drawbacks. One of the most significant disadvantages of VPS is the patient’s lifelong dependence on an implanted device. Once the shunt is placed, the patient becomes reliant on its continued functionality to maintain normal CSF dynamics. If the shunt fails—due to a blockage, disconnection, or mechanical malfunction—the patient may experience a rapid return of iNPH symptoms, such as gait instability and cognitive decline, necessitating immediate medical attention. The risk of shunt failure is substantial, with studies reporting that up to 40% of VPS patients require at least one shunt revision during their lifetime [9,41,42,43,44,45,46]. Revision surgeries are costly, invasive, and increase the patient’s risk of infection, anesthesia-related complications, and further surgical trauma. Infection is another critical concern with VPS, as the introduction of a foreign device into the body creates an opportunity for bacterial colonization. Shunt infections can be difficult to treat, often requiring removal of the device, intravenous antibiotic therapy, and subsequent re-implantation. In some cases, infection-related complications result in prolonged hospital stays and increased healthcare costs. VPS also carries a risk of over-drainage, which occurs when too much CSF is drained from the brain’s ventricles, resulting in subdural hematomas. This complication is particularly concerning for elderly patients, whose brains are more susceptible to the effects of over-drainage due to brain atrophy. Over-drainage can result in significant neurological deficits and may necessitate emergency intervention. Additionally, VPS patients require long-term follow-up to monitor shunt function and detect potential malfunctions. Unlike ETV, which is often a “one-and-done” procedure, VPS requires continuous oversight and lifetime device monitoring [9,41,42,43,44,45,46]. These follow-ups place a financial and logistical burden on both patients and healthcare systems, as they involve regular check-ups, radiological evaluations, and emergency revisions when problems arise.

Both ETV and VPS have clear advantages and disadvantages that must be carefully weighed when selecting the appropriate treatment for a patient with iNPH. ETV offers a long-term, device-free solution with a lower risk of infection and no dependency on implantable components. However, it is limited by patient anatomy, technical complexity, and the possibility of stoma closure. VPS, on the other hand, is a more versatile option that can be used in all iNPH patients, regardless of anatomical constraints, and provides more immediate symptom relief. However, it comes with the burden of lifelong device dependence, a higher risk of infection, and the possibility of multiple shunt revisions.

The choice between ETV and VPS should be made based on the patient’s individual anatomical characteristics, medical history, and clinical goals. By understanding the strengths and limitations of each approach, clinicians can make informed decisions that optimize patient outcomes and reduce the risk of postoperative complications.

### 4.2. Future Research Fields

The findings of this study will lay the groundwork for several future research directions aimed at advancing iNPH diagnosis, treatment, and rehabilitation. One promising area is the exploration of personalized medicine approaches to tailor surgical interventions based on patient-specific anatomical, physiological, and genetic factors. Advanced imaging techniques, such as high-resolution tractography and functional MRI, could be further developed to predict surgical responsiveness and optimize preoperative planning. Additionally, the role of neuroplasticity-promoting interventions, such as repetitive transcranial magnetic stimulation (nTMS), warrants deeper investigation. nTMS could be integrated into post-surgical rehabilitation protocols to enhance motor and cognitive recovery, particularly in patients with suboptimal surgical outcomes. The application of artificial intelligence (AI) and machine learning to analyze neuroimaging and clinical data presents another avenue for innovation. AI-driven models could identify novel biomarkers of iNPH and provide predictive analytics for long-term surgical outcomes. Moreover, longitudinal studies examining the durability of ETV and VPS outcomes over decades are needed to assess their impact on aging-related changes, such as cognitive decline and frailty. Finally, the investigation of minimally invasive and next-generation CSF diversion techniques, such as bioengineered shunt systems or non-surgical alternatives, could revolutionize treatment paradigms, offering safer and more cost-effective solutions for iNPH management. These research fields hold the potential to transform the current understanding and treatment of iNPH, leading to improved outcomes and quality of life for patients.

## 5. Conclusions

This study has the potential to revolutionize the management of iNPH by offering conclusive, comparative evidence on the efficacy, safety, and long-term outcomes of ETV versus VPS. By providing comprehensive data on cognitive and motor recovery, structural brain changes, and patient quality of life, this study aims to set a new standard for evidence-based decision making in neurosurgical care. The insights gained from this study will have the potential to influence clinical guidelines, reduce healthcare costs, and improve the quality of life for patients with iNPH. If ETV proves to be superior or at least non-inferior to VPS, it could become the preferred surgical treatment for iNPH. This shift would mark a significant advancement in the management of one of the most misdiagnosed yet treatable forms of dementia.

## Data Availability

No new data were created.
